# High Influenza A Virus Infection Rates in Mallards Bred for Hunting in the Camargue, South of France

**DOI:** 10.1371/journal.pone.0043974

**Published:** 2012-08-27

**Authors:** Marion Vittecoq, Viviane Grandhomme, Jocelyn Champagnon, Matthieu Guillemain, Bernadette Crescenzo-Chaigne, François Renaud, Frédéric Thomas, Michel Gauthier-Clerc, Sylvie van der Werf

**Affiliations:** 1 Centre de Recherche de la Tour du Valat, Arles, France; 2 Maladies Infectieuses et Vecteurs: Ecologie, Génétique, Evolution et Contrôle, Unité Mixte de Recherche (Institut de Recherche pour le Développement/Centre National de la Recherche Scientifique/Université Montpellier) 5290, Montpellier, France; 3 Département de Virologie, Institut Pasteur, Unité de Génétique Moléculaire des Virus à ARN, Paris, France; 4 Centre National de la Recherche Scientifique Unité de Recherche Associée 3015, Paris, France; 5 Université Paris Diderot Sorbonne Paris Cité, Paris, France; 6 Office National de la Chasse et de la Faune Sauvage, Centre National d’Etudes et de Recherches Appliquées avifaune migratrice, Arles, France; University of Texas Medical Branch, United States of America

## Abstract

During the last decade, the role of wildlife in emerging pathogen transmission to domestic animals has often been pointed out. Conversely, far less attention has been paid to pathogen transmission from domestic animals to wildlife. Here, we focus on the case of game restocking, which implies the release of millions of animals worldwide each year. We conducted a 2-year study in the Camargue (Southern France) to investigate the influence of hand-reared Mallard releases on avian influenza virus dynamics in surrounding wildlife. We sampled Mallards (cloacal swabs) from several game duck facilities in 2009 and 2010 before their release. A very high (99%) infection rate caused by an H10N7 strain was detected in the game bird facility we sampled in 2009. We did not detect this strain in shot ducks we sampled, neither during the 2008/2009 nor the 2009/2010 hunting seasons. In 2010 infection rates ranged from 0 to 24% in hand-reared ducks. The 2009 H10N7 strain was fully sequenced. It results from multiple reassortment events between Eurasian low pathogenic strains. Interestingly, H10N7 strains had previously caused human infections in Egypt and Australia. The H10 and N7 segments we sequenced were clearly distinct from the Australian ones but they belonged to the same large cluster as the Egyptian ones. We did not observe any mutation linked to increased virulence, transmission to mammals, or antiviral resistance in the H10N7 strain we identified. Our results indicate that the potential role of hand-reared Mallards in influenza virus epizootics must be taken into account given the likely risk of viral exchange between game bird facilities and wild habitats, owing to duck rearing conditions. Measures implemented to limit transmission from wildlife to domestic animals as well as measures to control transmission from domestic animals to wild ones need to be equally reinforced.

## Introduction

During the last decade, awareness concerning the intimate links between human and animal health has rapidly increased in the context of disease emergence [1]. Indeed, approximately 80% of the infectious diseases that recently emerged were zoonotic [2]. The role of wildlife in emerging pathogen transmission to humans and domestic animals has in many cases been pointed out [3]–[5]. Conversely, pathogen transmission from domestic animals to wildlife has received far less attention, although the importance of this issue was often mentioned [6], [7]. Indeed, contacts between wildlife and livestock or their environment sometimes result in wildlife diseases with conservation issues [8], [9]. Besides, hand-reared animal releases into the wild for either conservation or exploitation purposes represent a particular case in which hand-reared individuals eventually share natural habitats with their wild congeners. In both cases such releases can dramatically influence disease dynamics in the surrounding wild animal populations [10]–[14].

In the present study we focused on the case of game restocking, which implies the release of millions of individuals worldwide each year [15]. Birds are the most frequently involved, with millions of individuals being released annually in Europe only. For example more than 3 millions red-legged partridges are released annually in Spain [15], and ca. 1.4 million Mallards are being so in France [16]. The Camargue region, a complex network of wetlands situated in the Rhone delta, is a major duck winter quarter [17] and a central place for wildfowl hunting in France [18]. Hunting is also among the most important economic activities in the area, which is one of the reasons for the massive Mallard releases in the Camargue [19]. At least 30 000 hand-reared individuals are released annually in the region [20]. Maximum Mallard numbers in the wild are reached in September after the beginning of the hunting season, with 56 500 individuals on average over the last seven years (Gauthier-Clerc, unpubl. data), these numbers certainly include a mixing of wild and released Mallards. Given its central position on the flyway of many European migratory species, the Camargue is also a potential hotspot for the introduction and transmission of bird-borne pathogens [21].

For this reason, avian influenza viruses (AIV) have been studied since 2004 in the area. These negative-sense single stranded RNA viruses belonging to the *Orthomyxoviridae* family are commonly characterized by the combination of their surface proteins: hemagglutinin (HA) and neuraminidase (NA) [22], [23]. AIVs are highly variable and undergo continuous genetic evolution via two mechanisms: i) accumulation of point mutations at each replication cycle, ii) reassortment involving gene segment exchanges that occur when a cell is co-infected by different viruses [24]. These mechanisms contribute to the emergence of new variants with the ability to transmit to new hosts and/or with epidemic or even pandemic potential. Aquatic birds, particularly Anseriforms (ducks, geese and swans) and Charadriiforms (gulls, terns and shorebirds) constitute their major natural reservoir [23], [25]. The AIV circulating in wild birds are usually low pathogenic ones (LPAIV). LPAIV generally have little impact on their host [26], [27], although some studies have reported a possible influence on migration capacities [28]. Besides, when LPAIV of H5 or H7 subtypes are transmitted from wild birds to domestic ones reared in artificial environments, their virulence can evolve to high pathogenicity [29], [30]. Highly pathogenic avian influenza viruses (HPAIV), such as HP H5N1 strains currently circulating in Asia and Africa, are still of great economic concern, notably due to the cost of preventive actions including vaccination and massive birds culling [31]. Moreover, HPAIV infections represent a threat for human health since 603 HPAIV H5N1 human infections including 356 fatal cases have been reported worldwide since 2003 [32].

Relatively high prevalence of AIV was regularly detected in the wintering Mallard population of the Camargue (e.g. 5.4% prevalence during the 2006–2007 hunting season) [33]. Moreover a seasonal infection pattern was identified in Mallards during autumn and winter, with higher infection rates in early fall [33]. Mallards hence represent a focal study species in AIV research. Indeed wild Mallards are one of the main low pathogenic AIV natural reservoir host [34], and have proven to be healthy carriers of some of the H5N1 HPAIV strains [35]. However, to our knowledge no study ever aimed at investigating the potential role of hand-reared Mallards released for hunting in the epidemiology of AIV, despite the very large number of ducks being released in the wild annually.

To clear this gap we conducted a 2-year study in the Camargue to investigate the potential influence of hand-reared Mallard releases on AIV dynamics in surrounding wildlife. We first hypothesized that, owing to high density rearing conditions and to their genetic uniformity, hand-reared Mallards should be highly susceptible to AIV infections and could play an amplification role in AIV dynamics. This phenomenon has already been pointed out in red-legged partridge (*Alectoris rufa*) reared for hunting in Spain, where *Escherichia coli* prevalence was much higher in hand-reared populations before their release than in the wild ones [36]. To test this assumption we collected cloacal swabs from Mallards reared for hunting in several game bird facilities (GBF) in 2009 and 2010, and analysed these samples to measure AIV prevalence. Second, we hypothesized that AIV exchange occurs between wild and hand-reared Mallards, potentially leading either to the circulation of new strains in wild populations or to the amplification and dispersal of wild strains. Indeed, no barrier prevents AIV exchange between wild birds and hand-reared ducks in the GBF since water flows exist between pens and ponds used by wild birds. Moreover, as the GBF roofs are made of nets wild birds can deposit feces in the pens. Finally, hand-reared ducks are in direct contact with wild ones after their release. To investigate these issues, we tested shot waterfowl before and after hand-reared Mallards were sampled. Noteworthy, genetic analyses suggest that 76% of hunted Mallards in the Camargue have a captive origin [37]. Considering the low annual survival of released Mallards (0.8–15.9% depending on the release site) [37], the individuals we tested from the Camargue hunting bags certainly included a large proportion of ducks released some months before being shot. These released ducks cannot be differentiated morphologically from the wild ones [38]. Here we hence analyzed shot ducks as a whole since they are a representative sample of the Mallard population wintering in the Camargue, which is composed of individuals of both wild and captive origin that share habitats and can thus be considered as a single epidemiological unit.

Our third hypothesis was that any AIV strain potentially found in captive reared Mallards might present genetic characteristics linked to its circulation in domestic populations. We therefore performed a molecular study of the identified strains in order to look for such characteristics, and in particular to test for the presence of mutations known to be associated with increased virulence, since it has been highlighted that artificial environments are favorable to the appearance of such mutations in birds [30]. We also searched for mutations linked with transmission to other species including humans, since some studies proved that they can be acquired during their transmission among birds [39]. Thirdly, we tested for the presence of mutations conferring resistance to common antiviral drugs, since such resistance has recently been recorded in wild birds, notably in Sweden [40]. Lastly, full sequence analysis of some strains was performed, so as to get insight into their geographic origin through a phylogenetic study, and to determine their relatedness with strains, which have caused human infections in the past.

## Results

### Infection Rates and AIV Strains Involved

The infection rates in the different GBF were determined by real-time RT-PCR targeting the conserved M gene of AIV (Table 1). A very high infection rate (99%) was observed in the single GBF sampled in 2009. In 2010 the infection rates ranged between 0 and 24%, being of 8% in the farm infected in 2009. Initial subtyping, searching for H5, H7, H9, N1, and N7 by real-time RT-PCR performed on positive samples led to the identification of an H10N7 strain in the GBF in 2009 and further testing for H10 by real-time RT-PCR showed that the outbreak involved a single H10N7 virus (named H10N7 Camargue below). The viral strains involved in the infections observed in 2010 were LPAIV. H5, H7, H9, H10, and N7 subtypes were searched among them but none was detected.

**Table 1:** AIV infection rates in hand-reared ducks.

Year	GBF	Number of samples	Duckling age (weeks)	Days before release	AIV prevalence (%)
2009	GBF1	222	6	21	99
2010	GBF1	99	6	2	8
	GBF2	100	7	0	0
	GBF3	100	12.5	0	24
	GBF4	100	8	0	0

In shot ducks, mean AIV infection rates were 15% and 5% during the 2008/2009 and the 2009/2010 hunting seasons, respectively (Table 2). During these two periods the maximum monthly infection rates (respectively 20% and 16%) were observed in September (Table 2). All the involved strains were LPAIV but an important proportion of these were of the H5 subtype (2008/2009∶17%; 2009/2010∶30%). No H7 or H10N7 subtype was detected in AIV found in shot ducks.

**Table 2:** AIV infection rates in shot ducks.

	2008/2009		2009/2010	
Month	Number of samples	AIV prevalence (%)	Number of samples	AIV prevalence (%)
Sep.	170	20	97	16
Oct.	71	4	166	2
Nov.	22	18	139	2
Dec.	1	0	88	8
Jan.	1	0	63	0

### H10N7 Camargue Strain

#### Phylogeny

**Figure 1:**
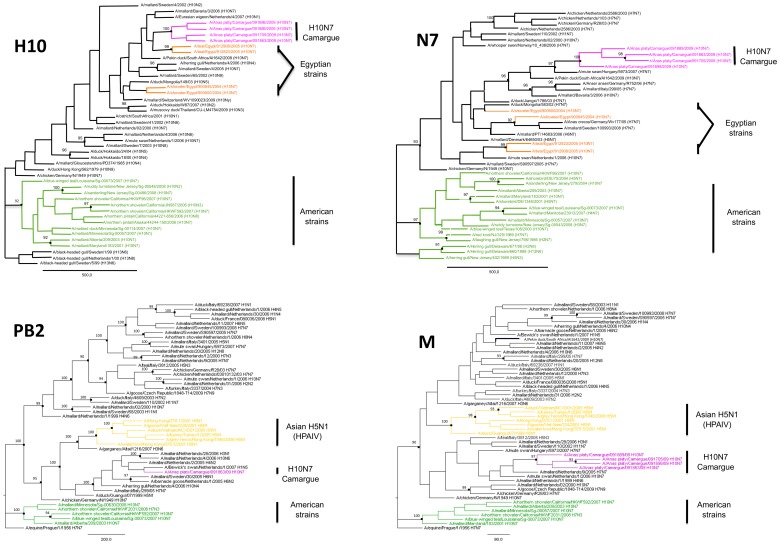
Phylogenetic trees of H10N7 Camargue viruses. ML phylogenies reconstructed from sequences of the H10, N7, PB2 and M segments. Topological supports summarized from 100 ML bootstrap replications are shown when ≥90. H10N7 Camargue viruses are in purple. Egyptian strains are shown in orange. American viruses are in green. Asian H5N1 highly pathogenic avian influenza strains are in yellow.

The H10N7 Camargue strain was isolated in embryonated chicken eggs and amplified. Determination of the sequence of the whole genome was performed in order to gain insights into its phylogeny and molecular characteristics. The phylogenetic analysis of four H10N7 Camargue isolates confirmed that they were very closely related to each other and formed a cluster (Figures 1 and S1). Analysis of each of the sequences of the 8 viral segments highlighted some shared characteristics (Figures 1 and S1). First, the sequences of all segments were clearly distinct from those of LPAIV strains from the North-American lineage. Second, none were closely related to highly pathogenic viruses (H5N1 or H7N7) that circulated in Europe and Asia. However, the PB2 segment was more closely related to the Asian HP H5N1 cluster than to most of the European LPAIV strains we included in our phylogenetic analysis. This finding suggests an ancient Asian origin of this segment. Third, the sequences of all segments were closely related to Eurasian strains. Interestingly, the most closely-related Eurasian strains differed between segments. As an example the LPAIV strain A/mute swan/Hungary/5973/2007 (H7N7) was very closely related to the H10N7 Camargue virus for the N7 segment, but less so for the M segment and belonged to a distant phylogenetic group for the PB2 segment. In the same way, the LPAIV strain A/mallard/Netherlands/9/2005 (H7N7) was closely related to the H10N7 Camargue virus for the M segment, whereas its PB2 segment belonged to a distinct phylogenetic group. African strains that we included in our analysis were part of Eurasian clusters and clearly distinct from American strains. For one of them, A/Pekin duck/South Africa/AI1642/2009 (H10N7) the NS, N7 and H10 sequences were closely related to those of the H10N7 Camargue virus but available sequences of two other segments (NP and M) were clearly distinct. Finally, it is important to note that based on the analysis of partial sequences of the H10 (data not shown) the H10N7 strains that caused human infections in Australia (Genebank accession numbers ADG58106 and ADG58107; Ratnamohan,V.M. and Dwyer, D.E. unpublished) are clearly distinct from the H10N7 Camargue strain. On the contrary Egyptian H10N7 strains that circulated in birds in 2004, i.e. at the time of the detection of two human infections [41], are relatively closely related to the H10N7 Camargue strain. However, this relatedness is difficult to interpret since the involved nodes are not strongly supported statistically (Figure 1).

#### Study of the extremities of the viral segments

We also determined the sequences of the extremities including the full non-coding sequences of all segments of the H10N7 Camargue strain, which to our knowledge was not performed before for any H10N7 strain. The extremities of the N7 segment revealed two characteristics that confirmed the phylogenetic distinction between the H10N7 Camargue strain and those of the American lineage. First, in the non-coding region of the 5′ extremity, an A was found at position 1440, as commonly seen in Eurasian strains, while strains of the American lineage possess a T at this position. Second, at position 145–147 of the 3′ extremity that belongs to the coding region the Camargue strain, like other Eurasian ones, has a supplementary amino acid compared to strains of the American lineage.

#### Molecular characterization

The molecular analysis of the H10N7 Camargue strain is detailed in Table 3. The focus strain is a low pathogenic one since the HA segment possesses a monobasic cleavage site. HA, NA and PB2 sequences all presented some typical avian characteristics (see Table 3). No known determinants of adaptation to mammals (humans or mice) were detected. In addition, we did not identify any mutation known to be linked with increased virulence in mammals. Yet, we detected the V149A mutation in PB2, which is associated with high virulence in chickens. However, 149A was also observed in low pathogenic H7 strains sampled from wild and domestic ducks in China. This suggests that this mutation alone does not increase the virulence in ducks [42]. Furthermore, no mutation was detected in the H10N7 Camargue strain at positions known to be linked with reduced susceptibility or resistance to neuraminidase inhibitors or M2-blockers.

**Table 3:** Molecular characteristics of the H10N7 Camargue strain. Amino acid substitutions related to known phenotypic traits identified for the H10N7 Camargue strain are in bold.

Characteristics/Phenotype	Segment	Sequence/Substitution	Reference	H10N7 Camargue
High pathogenicity	HA	RERRRKKR	[60]	Absent
**Typical avian RBS**	**HA**	**138A, 190E, 194L, 225G, 226Q, 228G (H3 numbering)**	[**61**]	**Present**
Adaptation to human α2,6 receptor	HA	H183N	[62]	Absent
**High hemadsorption activity (typical of avian viruses)**	**NA**	**367S, 370S, 372S, 400N, 403W, 432K**	[**63]**, [64****]	**Present except for the 432 position (432E)**
**Binding specificity for avian sialic acids**	**NA**	**275I, 431P**	[**65**]	**Present**
Mammalian Host range determinant	PB2	D701N	[66]	Absent
Mammalian Host range determinant	PB2	E627K	[67], [68]	Absent
High virulence in pigs	PB2	D92E	[69]	Absent
**High virulence in chickens**	**PB2**	**V149A**	[**70**]	**Present**
**Typical avian motif**	**PB2**	**Motif ESEV on the C-terminal position**	[**71**]	**Present**
Amantadine resistance	M2	A30T	[72]	Absent
Amantadine resistance	M2	S31N	[73]	Absent
Amantadine resistance	M2	G34A	[74]	Absent

## Discussion

### AIV Infections in Hand-reared Mallards

This study confirms our first hypothesis that hand-reared Mallards are highly sensitive to AIV infections. Indeed, we detected high infection rates in the GBF we sampled in 2009 (99%) and in 2010 (up to 24%), whereas the prevalence usually observed in duck populations very rarely exceeds 20% in the wild [33], [43], [44]. This shows that massive outbreaks can occasionally affect GBF, potentially impacting on AIV dynamics in the wild populations with which hand-reared Mallards are in contact before and after their release into the wild. This pattern has already been highlighted in Spain where rabbit (*Oryctolagus cuniculus*) restocking caused sarcoptic mange dispersion in the wild rabbit population, leading to massive decrease in numbers [11]. The possible outcomes of GBF infection onto wild duck populations should here depend on the origin of the strain involved. Indeed, low pathogenic AIV strains that naturally circulate in wild ducks have little impact on their health [26]. Our data did not permit to determine the origin of the strain we identified in 2009. Indeed, we did not detect the H10N7 Camargue strain among the samples collected on shot Mallards, neither during the hunting season that preceded the release of the infected individuals, nor during the following one.

### AIV Exchanges between Hand-reared and Wild Ducks

**Figure 2:**
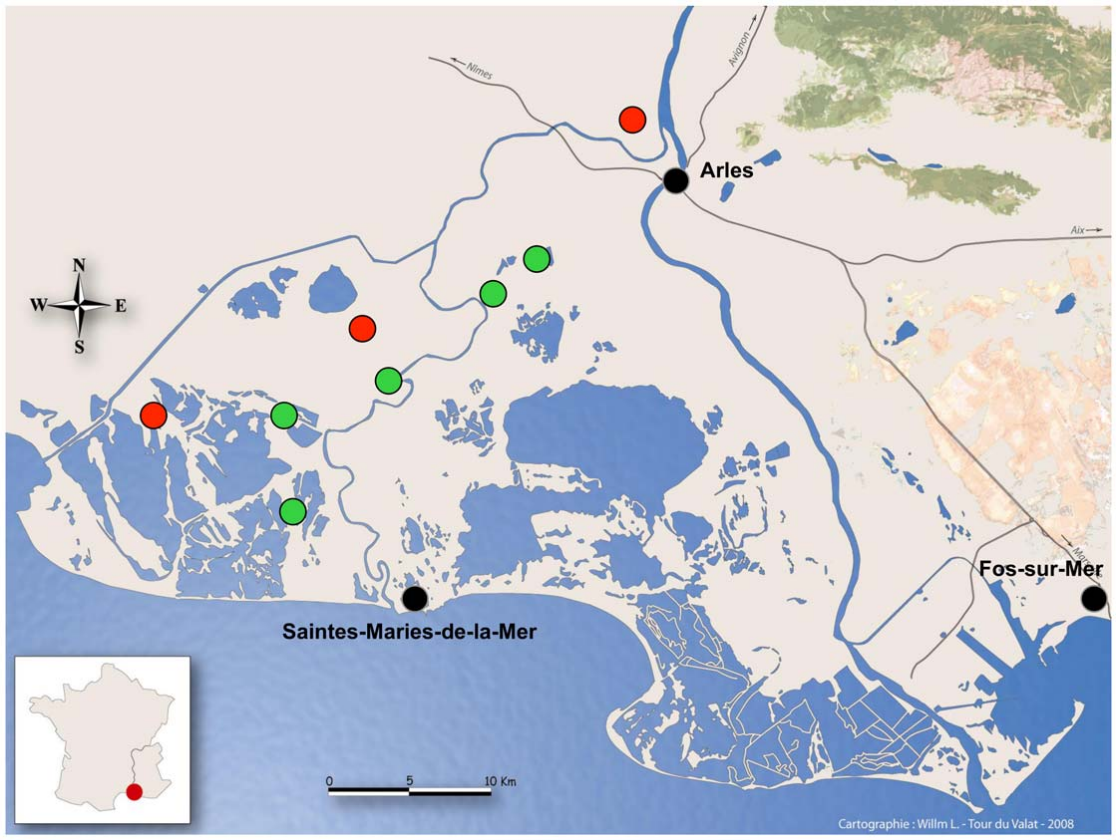
Situation of the sampling sites. Green circles represent the hunting estates where dead Mallards were sampled. Red circles represent the game bird facilities. One of the rearing estate (further named GBF3) is not represented since it is situated 50 km east from Arles, but the Mallards were sampled just before their release in several ponds of Fos–sur–Mer.

Our sample size for hunted Mallards was reasonably large (n = 299 in 2008/2009 and 555 in 2009/2010). Moreover, the sites where we sampled hand-reared and shot Mallards were close to each other (see Figure 2; for instance one of the hunting estates we sampled was within 4 km of the GBF where we detected an AIV outbreak). Nevertheless, our sampling effort was limited in time to the hunting seasons: we do not have any data from February to August 2009. We therefore cannot exclude that the H10N7 strain originated from wild birds. Population density during the breeding season is low in wild Mallard [45], and thus, even if the H10N7 Camargue virus originated from wild birds, hand-reared Mallards would then have played an amplifying role. In addition, commercial trade of Mallard chicks is common and most of the juveniles sampled in this study traveled 600 km from the producer when one-day old. It is also possible that the H10N7 Camargue strain originated from the birthplace of the chicks. If so, domestic ducks would have both played an amplifying role and potentially a dispersal one, since they could have spread a new strain in wild duck populations.

The lack of samples during the weeks that directly followed the release of the positive Mallards (because the hunting season had not yet started) could explain why we did not detect the H10N7 Camargue virus in wild individuals. Furthermore, the lack of large-scale dispersal of that H10N7 strain could be due to different parameters. First, we sampled the hand-reared ducks of the GBF1 21 days before their release. It is possible that only few birds were still excreting viruses when released into the wild, since excretion time in birds can be as short as 2 days (although it can also last up to 30 days in some cases) [46]–[48]. Second, a parallel capture-recapture demographic study ran in the Camargue has shown that hand-reared Mallards exhibit low monthly survival before the hunting season (only 44% survive from release until the onset of the hunting period on average) [49]. Moreover, their dispersal capacities appear to be very low [49]. The same pattern has been observed for red-legged partridges released in Spain [13]. Although intestinal parasites were much more numerous in the individuals living in the hunting estates where domestic birds were released, this was interestingly not observed in the neighboring estates [13]. It is possible that the poor survival and low dispersal of the hand-reared Mallards after release limited the potential spread of the H10N7 Camargue virus within the Mallard population in the wild.

Besides, we did not detect any H5 subtype circulating in the GBF whereas 23% of all the AIV we isolated from samples collected on shot Mallards in the Camargue belonged to this subtype. Again we could not highlight any AIV exchange between ducks living in captivity and in the wild. Yet, hand-reared ducks are exposed to wild bird feces and GBF are connected to wild bird habitats through common use of water. Thus, AIV exchanges are very likely to exist. Our results highlighted that: i) GBF represent an epidemiological compartment into which important AIV outbreaks can occur; ii) a significant proportion of Mallards wintering in the Camargue are infected by LPAIV, including H5 strains that are known to be able to evolve to HPAIV in domestic birds [30]. Knowing that rearing conditions in the GBF may favor AIV exchanges between wild and hand-reared Mallards, these findings outline the need for influenza surveillance in GBF to prevent HPAIV outbreaks in both wild and domestic birds in the region.

### Geographical Origin of the H10N7 Camargue Strain

We used the phylogenetical analysis of whole-genome sequence of the H10N7 Camargue strain as a supplementary tool to get insights into its evolutionary and geographical origins. The four isolates studied clearly belonged to a cluster that did not include other strains. The infections we detected were therefore certainly due to a single introduction event. All the viral segments of the H10N7 Camargue strain belong to the Eurasiatic lineage also including African strains. The sequences of the extremities of the N7 segment, that exhibit features characteristic of the Eurasian lineage viruses, confirmed this finding. The Eurasian lineage is clearly distinct from the American lineage and our observations further support the fact that inter-hemispheric AIV exchanges are rare. The Camargue strain was also distinct from the H10N7 viruses that previously caused human infections in Australia, but belonged to the same large cluster as the Egyptian H10N7 strains that circulated in birds when H10N7 human infections occurred in this country. These Egyptian strains did not cause severe disease. Nevertheless, this information raises concern about a possible transmission to humans of strains such as the Camargue one, in particular to the GBF owners and hunters who live in close contact with ducks. Interestingly, the strains that are genetically closest relatives differ for each segment of the H10N7 Camargue strain. The Camargue strain thus seems to result from multiple reassortments between different Eurasiatic strains, a common phenomenon which has been illustrated by many studies [50], [51]. Regrettably, we could not determine if the H10N7 Camargue strain was more closely related to strains observed in the wild or in GBF. Indeed, the data associated with viral sequences from AIV infected Mallards in GenBank are often too scarce to determine if the sample was taken on a wild or a domestic individual. This underlines the need for more detailed information on AIV sequences included in common databases.

### Molecular Characteristics of the H10N7 Camargue Strain

The molecular analysis of the H10N7 Camargue strain allowed us to question whether it may exhibit some characteristics related to its circulation in an artificial environment, namely some mutations linked to increased virulence, resistance to antiviral drugs or transmission to humans. Our results do not support this assumption. The sequences of the studied segments presented typical avian characteristics. No mutation known to contribute to transmission to humans was detected. Moreover, the Camargue strain is a low pathogenic one like all viruses isolated during our research program in the Camargue since 2005 [33], [52]. No mutation conferring antiviral resistance was detected. This analysis therefore failed to highlight any risk factor linked to this particular strain. Yet, one must keep in mind that we only looked for mutations previously described in the literature, while many others may remain undiscovered so far. It is also important to stress that most experimental studies focusing on mutations modifying host specificity and virulence are carried out on chicken, although it has been demonstrated that phenotypic outcomes of a given mutation can greatly differ from one host species to another [42].

In conclusion, our results point out the important role that could be played by hand-reared ducks released for hunting in AIV dynamics. As we did not detect similar AIV strains in shot and hand-reared ducks we could not prove that AIV exchanges exist between these two epidemiological compartments. Yet, due to rearing conditions in GBF, AIV exchange risks seem to be high enough to urge for sanitary control of hand-reared animals prior to their release into the wild, which appears to be highly insufficient so far. Such surveillance would also prevent HPAIV circulation that may arise from the evolution in GBF of H5 LPAIV that proved to be commonly infecting free-living ducks in the Camargue. The world organization for animal health (OIE) stresses that surveillance of AIV infection should be applied to all domesticated birds including those used “for restocking supplies of game” [53], but control measures generally appear to be poor in game bird rearing estates. This surveillance gap has recently been highlighted in the USA, where game bird holders reported very variable sanitary practices [54]. The problem appears similar in Europe, including France. Additionally, according to French law, game birds should be ringed to allow for the differentiation of wild and released individuals [55]. Most of GBF owners ignore this obligation. If released birds were clearly identified the specific role they may have in AIV dynamics could be addressed both before and after their release. Our study illustrates the reality of the epidemiological consequences that can result from surveillance gaps and the knowledge that could be gained if released individuals were recognizable. Sharing knowledge and strictly controlling viral exchanges between wild birds and all kinds of domestic ones represent major steps to anticipate and face HPAIV epizootics.

## Methods

### Ethics Statement

This study has been submitted for approval and the results reviewed by the Scientific Council of the Tour du Valat Foundation. Birds were handled, ringed and sampled under the supervision of a veterinarian (Michel Gauthier-Clerc) and a registered duck ringer of the « Museum National d’Histoire Naturelle » of Paris (Matthieu Guillemain). All procedures were carried out in accordance with the permit delivered by the prefect of Paris to the « Office National de la Chasse et de la Faune Sauvage » (permit n 2009–014).

### Bird Sampling

Cloacal swabs were taken in the Camargue between August 2008 and July 2010 to collect faecal samples from hand-reared Mallards and wild ducks (see sampling sites in Figure 2). Using a network of hunters, 299 and 552 samples were taken on locally shot Mallards during the 2008/2009 and 2009/2010 hunting seasons (September to January), respectively. Samples were collected on hand-reared Mallards during the pre-release ringing sessions of these birds in one GBF in 2009 and 4 GBF (including the one sampled in 2009) in 2010. Ducks were juveniles (5 to 10 weeks old), reared in open-air and released immediately or within a few days or weeks after sampling (Table 1). Samples were stored in 3 ml of universal transport medium (Biolys kit) and frozen at −80°C until molecular analysis was performed.

### RNA Isolation and Virus Detection

RNA isolation from 140 µl of each sample was carried out using a Macherey-Nagel NucleoSpin 96 virus system with RNA elution into a final volume of 60 µl. Influenza A virus was detected by real-time RT-PCR targeting the conserved matrix as describe previously [56]. All real-time RT-PCR assays were performed on a LightCycler 480 (Roche Diagnostic) in a final volume of 10 µl with 2.5 µl RNA, 0.5 µM of each primer, 0.2 µM probe and 0.4 µl enzyme mix using a Superscript III Platinum One-Step quantitative RT-PCR system (Invitrogen). The reaction was carried out with the following temperature profile: 15 min at 45°C, 3 min at 95 C, 50 cycles of 10 s at 95°C, 10 s at 55°C, 20 s at 72°C, and finally 30 s at 40°C. Positive samples were further tested for H5, H7, H9, N1 and N7 subtypes using the same real-time RT-PCR technique (primers available upon request).

### Virus Isolation and Characterization

For 10 RT-PCR positive samples, 200 µl of specimen was inoculated in the allantoic cavity of 10-days-old embryonated hen’s eggs. The allantoic fluid was harvested after 72 h at 37°C and influenza A virus was detected by hemagglutination assay with hen erythrocytes. Viral RNA was purified from allantoic fluid using a QIAamp viral RNA mini kit (Qiagen) following manufacturer’s instructions. HA subtype of virus isolates was determined by RT-PCR and sequencing of the HA_0_ cleavage site region using a universal set of primers as described previously [57]. HA sequence was analyzed with the basic local alignment search tool available from NCBI (data not shown) and confirmed by sequencing of the whole HA gene using a set of H10-specific primers (primer sequences available upon request). The NA subtype was deduced from this analysis and confirmed by RT-PCR and sequencing of 172 nt of the NA using a set of H10-specific primers (primer sequences available upon request).

### Gene Sequencing and Phylogenetic Analysis

Amplification of viral RNA extracted from 4 virus isolates was carried out using a Superscript Platinum One-step RT-PCR system (Invitrogen) and primers specific of each segment. Sequencing was done using a Big Dye Terminator V1.1 kit and a sequencer ABI DNA Analyzer 3730XL (Applied Biosystems). Sequences of all primers are available upon request. Sequences of the whole viral genome were analyzed with CLC Main Workbench 5.6.1. We performed alignments for the 8 segments using sequences available from the Influenza Sequence Database with CLC Main Workbench 5.6.1. Phylogenetic trees were constructed using maximum parsimony (MP) methods with the dnapars program of the PHYLIP 3.68 package and the maximum likelihood (ML) with the software PhyML 2.4.4. Evolutionary model was selected using Model Generator 0.85 [58]. Nodal supports were assessed with 100 bootstrap replicates generated for each method.

### Determination of 3′ and 5′ End Non Coding Sequences of the 8 Segments of the H10N7 Virus

Viral genomic RNA was extracted using the QIAamp Viral RNA Mini kit (Qiagen) from 140 µl of allantoic fluid according to the manufacturer’s recommendations. The RNA was eluted in 60 µl of RNase-free water and the 3′ and 5′ NC regions were amplified as previously described [59] using an anchored (dT)_14_ oligonucleotide and primers specific for the coding sequences of both segments for reverse transcription and amplification. After purification, the PCR products were sequenced with internal oligonucleotides using a Big Dye terminator sequencing kit and an automated sequencer (Perkin Elmer). All sequences of the primers are available from the authors upon request.

### Rapid Molecular Diagnostic

One-step real-time RT-PCR assays were developed to be specific of the virus identified. All influenza A positive samples detected between August 2008 and July 2010 on wild and domestic Mallards were tested for H10 and N7 subtypes respectively. Detection was performed in the same conditions as described above except annealing temperature was decreased from 55°C to 50°C using the following primers and probes: H10-780Fw (5′-TCT-GAT-AGC-ACC-GAG-CC-3′) and H10-888Rv (5′-ACC-CCC-TCT-CCA-AAA-AC-3′) primers and H10-832+MGB 5′ [6Fam]-CAA-TCA-GAC-GCA-CCA-AT-[MGBNFQ]3′) probe specific of H10 subtype; N7-1260Fw (5′-CAA-TCC-ATG-CTT-TTA-CGT-AG-3′) and N7-1334Rv (5′-CTG-TTA-CTT-GTC-CAC-CAT-3′) primers and N7-1306Probe(-) (5′ [Fam]-CCT-CTT-CGG-GTC-TTC-CTC-T-[BHQ-1]3′) probe specific of N7 subtype.

## Supporting Information

Figure S1 **Phylogenetic trees of H10N7 Camargue viruses.** ML phylogenies reconstructed from sequences of the NP, PA, NS and PB1 segments. Topological supports summarized from 100 ML bootstrap replications are shown when ≥90. H10N7 Camargue viruses are in purple. American viruses are in green. Asian H5N1 highly pathogenic avian influenza strains are in yellow.
(TIF)
