# Supplementary figures and images for: High Influenza A Virus Infection Rates in Mallards Bred for Hunting in the Camargue, South of France
Source: PLoS One. 2012 Aug 27;7(8):e43974. doi: 10.1371/journal.pone.0043974 (PMC3428329; doi:10.1371/journal.pone.0043974)

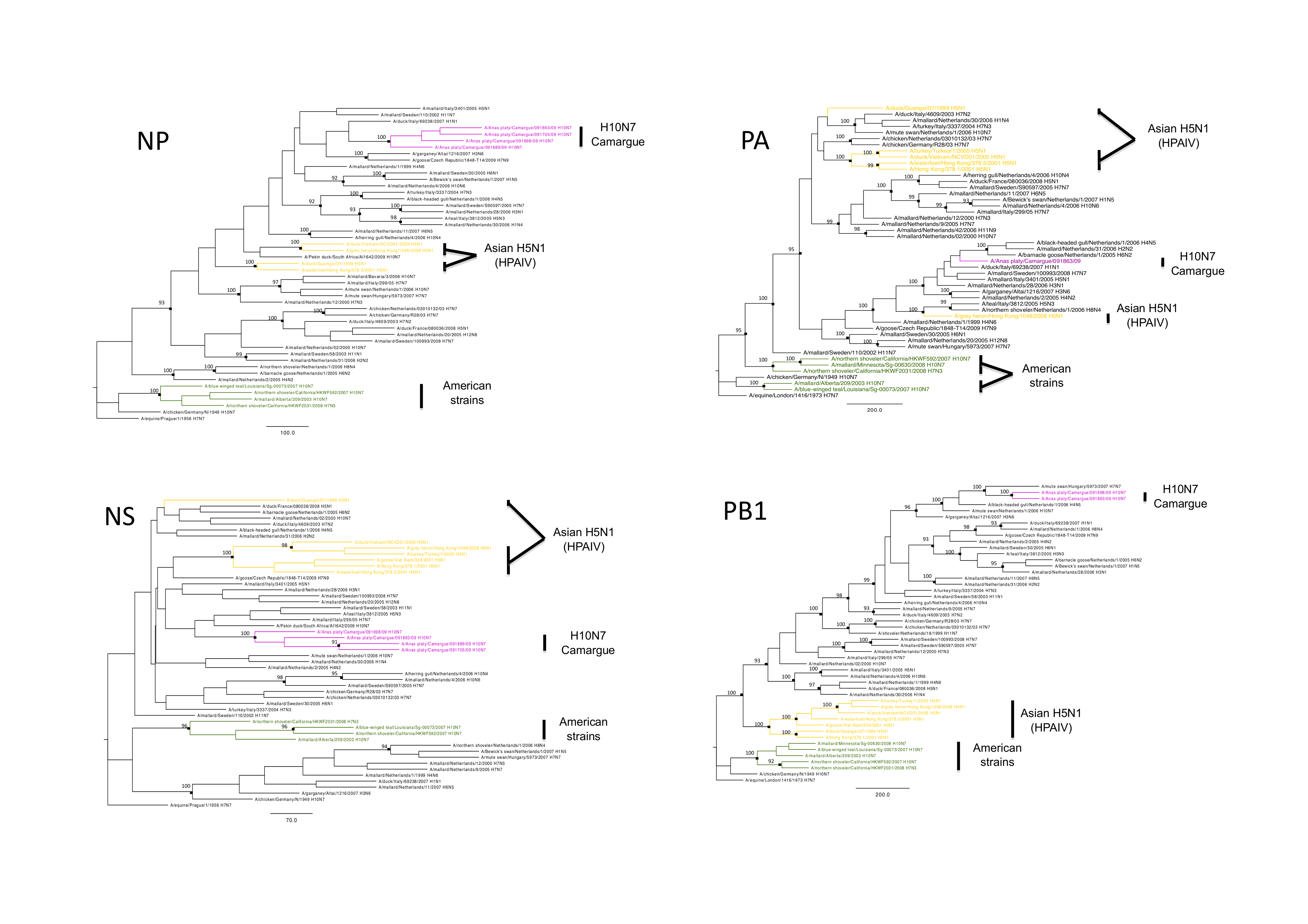

Supplement: Figure S1 — Phylogenetic trees of H10N7 Camargue viruses. ML phylogenies reconstructed from sequences of the NP, PA, NS and PB1 segments. Topological supports summarized from 100 ML bootstrap replications are shown when ≥90. H10N7 Camargue viruses are in purple. American viruses are in green. Asian H5N1 highly pathogenic avian influenza strains are in yellow. (TIF) [file pone.0043974.s001.tif]
